# Supplementary material for: Composable languages for bioinformatics: the NYoSh experiment
Source: PeerJ. 2014 Jan 2;2:e241. doi: 10.7717/peerj.241 (PMC3898313; doi:10.7717/peerj.241)
Supplement: Figure S1 [file peerj-02-241-s001.pdf]

File - script.sh

```
1  # This is the only function that aligners need to implement.
2  # Parameters:
3  #   $1: a temporary filename
4  #   $2: the basename that should be used to store the sorted alignment
5
6  function plugin_align {
7    #sample parameters reading
8    OUTPUT=$1
9    BASENAME=$2
10   #invoke the model through the script generated by the GobyWeb language
11   . ${JOB_DIR}/run_model.sh plugin_align ${OUTPUT} ${BASENAME}
12 }
13
14
```

```
 1
 2  export MPS_HOME=${RESOURCES_ARTIFACTS_MPS_DISTRIBUTION}
 3  MPS_LIBS=`cat ${RESOURCES_MPS_JARS_LIST} |awk '{ORS=":"; print $1}'`
 4  NYOSH_SUPPORT_LIBS="$RESOURCES_ARTIFACTS_MPS_SUPPORT_LIBS/*"
 5  CLASSPATH=${MPS_LIBS}${NYOSH_SUPPORT_LIBS}:${JOB_DIR}/plugin.jar:${JOB_DIR}
 6  MODEL=BWAGobyArtifactPlugin
 7  NYOSH_SCRIPTNAME=BWAGobyArtifactScript
 8  CLASSNAME=${MODEL}.${NYOSH_SCRIPTNAME}
 9  java ${PLUGIN_NEED_DEFAULT_JVM_OPTIONS} -classpath ${CLASSPATH} ${CLASSNAME} "$
    @"
10
11
```

```java
package BWAGobyArtifactPlugin;

/*Generated by MPS */

import org.campagnelab.nyosh.logging.StepsLoggerHelper;

public class ErrorManagementImplementation {
    public void recordStepDone(String actionDescription) {
        StepsLoggingSuccessHandler_ym50rj_(actionDescription);

    }

    public void exception(String actionDescription, int statusCode, Exception exception) {
        StepsLoggingErrorHandler_kysnd9_(actionDescription, statusCode, exception);
    }

    public static void StepsLoggingErrorHandler_kysnd9_(String actionDescription, int statusCode, Exception exception) {
        StepsLoggerHelper.createLogFile();
        StepsLoggerHelper.assertTrue(false, "A step failed");
    }

    public static void StepsLoggingSuccessHandler_ym50rj_(String actionDescription) {
        StepsLoggerHelper.createLogFile();
        StepsLoggerHelper.done(actionDescription, 0);
    }

}
```

```
1  package BWAGobyArtifactPlugin;
2
3  /*Generated by MPS */
4
5  import java.util.Set;
6  import java.util.HashSet;
7
8  import org.campagnelab.nyosh.environment.parsers.Parser;
9  import org.campagnelab.nyosh.environment.parsers.JVMEnvParser;
10
11 import java.util.SortedSet;
12
13 import org.campagnelab.nyosh.environment.parsers.ScriptVariable;
14 import org.campagnelab.nyosh.environment.parsers.GobyWebParser;
15 import org.campagnelab.nyosh.environment.NYoShRuntimeEnvironment;
16 import org.apache.commons.io.FilenameUtils;
17 import org.campagnelab.nyosh.environment.parsers.MapFileParser;
18 import org.campagnelab.nyosh.exec.CommandAssembler;
19 import org.campagnelab.nyosh.exec.CommandExecutionPlan;
20 import org.campagnelab.stepslogger.FileStepsLogger;
21
22 import java.io.File;
23 import java.io.IOException;
24
25 import org.apache.log4j.Logger;
26 import org.apache.log4j.LogManager;
27
28 public class BWAGobyArtifactScript {
29
30     private static Set<String> exportedVariables = new HashSet<String>();
31
32     public static void main(String[] arguments) {
33         if (arguments.length == 0) {
34             arguments = new String[]{"main"};
35         }
36
37         // BEFORE_ENTRY_POINT_EXECUTION
38
39         if ("plugin_align".equals(arguments[0])) {
40
41             if (arguments.length == 3) {
42                 align(arguments[1], arguments[2]);
43             } else {
44                 System.err.println("Invalid number of arguments");
45             }
46             finish();
47             System.exit(0);
48         }
49         System.err.printf("The entry point %s name was not recognized", arguments[0]);
50         finish();
51         System.exit(1);
52     }
53
54     public static void align(String output, String basename) {
55         {
56             initializeStepsLogging();
57             System.out.println("Executing step: " + "Catch all steps for GobyWeb");
58             boolean success_u4s4ck_a0d = false;
59             String reason_u4s4ck_a0d = "Catch all steps for GobyWeb";
60             Exception exception_a0d = null;
61             try {
62                 {
63                     Parser parser_u4s4ck_a0a0a0a5a0a0a0d = new JVMEnvParser();
64                     SortedSet<ScriptVariable> variables_u4s4ck_a0a0a0a5a0a0a0d = parser_u4s4ck_a0a0a0a5a0a0
    a0d.parseAtRunTime();
65                     Parser parser_u4s4ck_b0a0a0f0a0a0a3 = new GobyWebParser();
```

```
66                  SortedSet<ScriptVariable> variables_u4s4ck_b0a0a0f0a0a0a3 = parser_u4s4ck_b0a0a0f0a0a0
    a3.parseAtRunTime();
67
68                  String COLOR_SPACE_OPTION = (NYoShRuntimeEnvironment.getEnvironment().getVar
    iableValue("COLOR_SPACE").equals("true") ?
69                          "-c" :
70                          ""
71                  );
72                  String BWA_GOBY_EXEC_PATH = NYoShRuntimeEnvironment.getEnvironment().getVa
    riableValue("RESOURCES_ARTIFACTS_BWA_WITH_GOBY_ARTIFACT_EXECUTABLE") + ;
73                  String ORG = NYoShRuntimeEnvironment.getEnvironment().getVariableValue("
    ORGANISM").toUpperCase();
74                  System.out.println("Genome reference id: " + NYoShRuntimeEnvironment.getEnvironme
    nt().getVariableValue("GENOME_REFERENCE_ID"));
75                  String[] genomeInfo = NYoShRuntimeEnvironment.getEnvironment().getVariableValue("
    GENOME_REFERENCE_ID").toUpperCase().split("\\.");
76                  String BUILD_NUMBER = "";
77                  String ENSEMBL_RELEASE = "";
78                  if (genomeInfo.length == 2) {
79                      BUILD_NUMBER = genomeInfo[0];
80                      ENSEMBL_RELEASE = genomeInfo[1];
81                  } else {
82                      fail(false, "Invalid genome " + NYoShRuntimeEnvironment.getEnvironment().getVaria
    bleValue("GENOME_REFERENCE_ID"), 1);
83                  }
84                  String SAMPE_SAMSE_OPTIONS =
85                          NYoShRuntimeEnvironment.getEnvironment().getVariableValue("
    PLUGINS_ALIGNER_BWA_GOBY_ARTIFACT_NYOSH_SAMPE_SAMSE_OPTIONS");
86                  String ALL_OTHER_OPTIONS =
87                          NYoShRuntimeEnvironment.getEnvironment().getVariableValue("
    PLUGINS_ALIGNER_BWA_GOBY_ARTIFACT_NYOSH_ALL_OTHER_OPTIONS");
88                  int BWA_GOBY_NUM_THREADS = 4;
89                  String SAMPLE_NAME = FilenameUtils.getBaseName(NYoShRuntimeEnvironment.getEn
    vironment().getVariableValue("READS_FILE"));
90                  String PLATFORM_NAME = NYoShRuntimeEnvironment.getEnvironment().getVariableV
    alue("READS_PLATFORM");
91                  String READ_GROUPS = "@RG\\tID:1\\tSM:" + SAMPLE_NAME + "\\tPL:" + PLAT
    FORM_NAME + "\\tPU:1";
92                  String INDEX_DIR_KEY = "
    RESOURCES_ARTIFACTS_BWA_WITH_GOBY_ARTIFACT_INDEX_" + ORG + "_" + BUILD
    _NUMBER + "_" + ENSEMBL_RELEASE;
93                  String INDEX_DIR = NYoShRuntimeEnvironment.getEnvironment().getVariableValue(IND
    EX_DIR_KEY) + "/index";
94                  System.out.println("Index directory is: " + INDEX_DIR);
95                  System.out.println("Loading environment from: "
96                          + NYoShRuntimeEnvironment.getEnvironment().getVariableValue("
    RESOURCES_ARTIFACTS_PROTOBUF_CPP_LIBRARIES")
97                          + "/setup.sh");
98                  MapFileParser parser_u4s4ck_a91a0a0a5a0a0a0d = new MapFileParser();
99                  SortedSet<ScriptVariable> variables_u4s4ck_a91a0a0a5a0a0a0d =
100                         parser_u4s4ck_a91a0a0a5a0a0a0d.parseAtRunTime(String.format("%s/%s",
101                                 NYoShRuntimeEnvironment.getEnvironment().getVariableValue("
    RESOURCES_ARTIFACTS_PROTOBUF_CPP_LIBRARIES"),
102                                 "setup.sh"));
103                 for (ScriptVariable var : variables_u4s4ck_a91a0a0a5a0a0a0d) {
104                     exportedVariables.add(var.name);
105                 }
106                 System.out.println("Loading environment from: "
107                         + NYoShRuntimeEnvironment.getEnvironment().getVariableValue("
    RESOURCES_ARTIFACTS_GOBY_CPP_API_LIBRARIES")
108                         + "/setup.sh");
109                 MapFileParser parser_u4s4ck_a12a0a0a5a0a0a0d = new MapFileParser();
110                 SortedSet<ScriptVariable> variables_u4s4ck_a12a0a0a5a0a0a0d =
111                         parser_u4s4ck_a12a0a0a5a0a0a0d.parseAtRunTime(String.format("%s/%s",
112                         NYoShRuntimeEnvironment.getEnvironment().getVariableValue("
    RESOURCES_ARTIFACTS_GOBY_CPP_API_LIBRARIES"),
```

```
113                         "setup.sh"));
114                 for (ScriptVariable var : variables_u4s4ck_a12a0a0a5a0a0a0d) {
115                     exportedVariables.add(var.name);
116                 }
117                 String SAI_FILE_0 = String.format("%s%s-0.sai",
118                         FilenameUtils.getFullPath(NYoShRuntimeEnvironment.getEnvironment().getVariable
    Value("READS_FILE")), SAMPLE_NAME);
119                 String SAI_FILE_1 = String.format("%s%s-1.sai",
120                         FilenameUtils.getFullPath(NYoShRuntimeEnvironment.getEnvironment().getVariable
    Value("READS_FILE")), SAMPLE_NAME);
121                 {
122                     StringBuffer commandBuffer = new StringBuffer();
123                     CommandAssembler assembler = new CommandAssembler();
124                     assembler.appendCommand("nice " + BWA_GOBY_EXEC_PATH
125                             + " aln -w 0 -t " + BWA_GOBY_NUM_THREADS
126                             + " " + COLOR_SPACE_OPTION + " -f "
127                             + SAI_FILE_0 + " -l "
128                             + NYoShRuntimeEnvironment.getEnvironment().getVariableValue("
    INPUT_READ_LENGTH")
129                             + " " + ALL_OTHER_OPTIONS + " -x "
130                             + NYoShRuntimeEnvironment.getEnvironment().getVariableValue("
    START_POSITION")
131                             + " -y "
132                             + NYoShRuntimeEnvironment.getEnvironment().getVariableValue("
    END_POSITION")
133                             + " " + INDEX_DIR + " " + NYoShRuntimeEnvironment.getEnvironment().getV
    ariableValue("READS_FILE"));
134                     commandBuffer.append("nice " + BWA_GOBY_EXEC_PATH + " aln -w 0 -t "
135                             + BWA_GOBY_NUM_THREADS + " " + COLOR_SPACE_OPTION + " -f "
136                             + SAI_FILE_0 + " -l "
137                             + NYoShRuntimeEnvironment.getEnvironment().getVariableValue("
    INPUT_READ_LENGTH")
138                             + " " + ALL_OTHER_OPTIONS + " -x "
139                             + NYoShRuntimeEnvironment.getEnvironment().getVariableValue("
    START_POSITION")
140                             + " -y "
141                             + NYoShRuntimeEnvironment.getEnvironment().getVariableValue("
    END_POSITION")
142                             + " " + INDEX_DIR
143                             + " "
144                             + NYoShRuntimeEnvironment.getEnvironment().getVariableValue("
    READS_FILE"));
145                     // process output according to type
146                     CommandExecutionPlan plan = null;
147                     lastExitCode = -1;
148                     try {
149                         assembler.setLocalEnvironment(exportedVariables);
150                         assembler.finishAssembly();
151                         plan = assembler.getCommandExecutionPlan();
152                         lastExitCode = plan.run();
153
154                     } finally {
155                         if (plan == null || !(plan.executedCompletely())) {
156                             errorManagement.exception("failed executing: " + commandBuffer.toString(), 0, n
    ull);
157                         } else {
158                             errorManagement.recordStepDone("successfully executed: " + commandBuffer.toS
    tring());
159                         }
160                     }
161                 }
162
163                 {
164                     StringBuffer commandBuffer = new StringBuffer();
165                     CommandAssembler assembler = new CommandAssembler();
166                     assembler.appendCommand("nice " + BWA_GOBY_EXEC_PATH
```

```
167                         + " aln -w 1 -t " + BWA_GOBY_NUM_THREADS
168                         + " " + COLOR_SPACE_OPTION + " -f "
169                         + SAI_FILE_1 + " -l "
170                         + NYoShRuntimeEnvironment.getEnvironment().getVariableValue("
    INPUT_READ_LENGTH")
171                         + " " + ALL_OTHER_OPTIONS + " -x "
172                         + NYoShRuntimeEnvironment.getEnvironment().getVariableValue("
    START_POSITION")
173                         + " -y "
174                         + NYoShRuntimeEnvironment.getEnvironment().getVariableValue("
    END_POSITION")
175                         + " " + INDEX_DIR + " "
176                         + NYoShRuntimeEnvironment.getEnvironment().getVariableValue("
    READS_FILE"));
177                     commandBuffer.append("nice " + BWA_GOBY_EXEC_PATH
178                         + " aln -w 1 -t " + BWA_GOBY_NUM_THREADS + " "
179                         + COLOR_SPACE_OPTION + " -f " + SAI_FILE_1
180                         + " -l "
181                         + NYoShRuntimeEnvironment.getEnvironment().getVariableValue("
    INPUT_READ_LENGTH")
182                         + " " + ALL_OTHER_OPTIONS + " -x "
183                         + NYoShRuntimeEnvironment.getEnvironment().getVariableValue("
    START_POSITION")
184                         + " -y "
185                         + NYoShRuntimeEnvironment.getEnvironment().getVariableValue("
    END_POSITION")
186                         + " " + INDEX_DIR + " "
187                         + NYoShRuntimeEnvironment.getEnvironment().getVariableValue("
    READS_FILE"));
188                     // process output according to type
189                     CommandExecutionPlan plan = null;
190                     lastExitCode = -1;
191                     try {
192                         assembler.setLocalEnvironment(exportedVariables);
193                         assembler.finishAssembly();
194                         plan = assembler.getCommandExecutionPlan();
195                         lastExitCode = plan.run();
196 
197                     } finally {
198                         if (plan == null || !(plan.executedCompletely())) {
199                             errorManagement.exception("failed executing: " + commandBuffer.toString(), 0, n
    ull);
200                         } else {
201                             errorManagement.recordStepDone("successfully executed: " + commandBuffer.toS
    tring());
202                         }
203                     }
204                 }
205 
206                 {
207                     StringBuffer commandBuffer = new StringBuffer();
208                     CommandAssembler assembler = new CommandAssembler();
209                     assembler.appendCommand("nice " + BWA_GOBY_EXEC_PATH + " sampe "
210                         + COLOR_SPACE_OPTION + " " + SAMPE_SAMSE_OPTIONS + " -F goby
     -f "
211                         + output + " -x "
212                         + NYoShRuntimeEnvironment.getEnvironment().getVariableValue("
    START_POSITION")
213                         + " -y "
214                         + NYoShRuntimeEnvironment.getEnvironment().getVariableValue("
    END_POSITION")
215                         + " " + INDEX_DIR + " " + SAI_FILE_0
216                         + " " + SAI_FILE_1 + " "
217                         + NYoShRuntimeEnvironment.getEnvironment().getVariableValue("
    READS_FILE")
218                         + " -r " + READ_GROUPS);
```

```
219                     commandBuffer.append("nice " + BWA_GOBY_EXEC_PATH
220                             + " sampe " + COLOR_SPACE_OPTION + " "
221                             + SAMPE_SAMSE_OPTIONS + " -F goby -f "
222                             + output + " -x "
223                             + NYoShRuntimeEnvironment.getEnvironment().getVariableValue("
    START_POSITION")
224                             + " -y " + NYoShRuntimeEnvironment.getEnvironment().getVariableValue("
    END_POSITION")
225                             + " " + INDEX_DIR + " " + SAI_FILE_0 + " "
226                             + SAI_FILE_1 + " "
227                             + NYoShRuntimeEnvironment.getEnvironment().getVariableValue("
    READS_FILE")
228                             + " -r " + READ_GROUPS);
229                     // process output according to type
230                     CommandExecutionPlan plan = null;
231                     lastExitCode = -1;
232                     try {
233                         assembler.setLocalEnvironment(exportedVariables);
234                         assembler.finishAssembly();
235                         plan = assembler.getCommandExecutionPlan();
236                         lastExitCode = plan.run();
237
238                     } finally {
239                         if (plan == null || !(plan.executedCompletely())) {
240                             errorManagement.exception("failed executing: " + commandBuffer.toString(), 0, n
    ull);
241                         } else {
242                             errorManagement.recordStepDone("successfully executed: " + commandBuffer.toS
    tring());
243                         }
244                     }
245                 }
246             }
247             success_u4s4ck_a0d = true;
248         } catch (Exception e) {
249             exception_a0d = e;
250
251         } finally {
252             if (!(success_u4s4ck_a0d)) {
253                 errorManagement.exception("step " + reason_u4s4ck_a0d + " failed.", 0, exception_a0d);
254             } else {
255                 errorManagement.recordStepDone(reason_u4s4ck_a0d);
256             }
257
258             try {
259                 // This was the last step, we need to close the stepslogger:
260                 _steps.close();
261
262             } catch (Exception e) {
263                 if (LOG.isInfoEnabled()) {
264                     LOG.info("An error occured closing stepslogger", e);
265                 }
266             }
267         }
268     }
269     // end of reduce_step
270 }
271
272 public static void finish() {
273 }
274
275 private static FileStepsLogger _steps;
276
277 // declared flag removed
278 public static void initializeStepsLogging() {
279     if (BWAGobyArtifactScript._steps == null) {
```

```
280             BWAGobyArtifactScript._steps = new FileStepsLogger(new File("./"));
281         }
282     }
283
284     public static void fail(boolean mustBeTrue, String reason) {
285         fail(mustBeTrue, reason, 1);
286     }
287
288     private static void done(String stepDescription, int statusCode) {
289         BWAGobyArtifactScript._steps.step(stepDescription, statusCode);
290     }
291
292     /*package*/
293     static void fail(boolean mustBeTrue, String reason, int statusCode) {
294         if (!(mustBeTrue)) {
295             BWAGobyArtifactScript._steps.error(reason);
296             try {
297                 BWAGobyArtifactScript._steps.close();
298             } catch (IOException e) {
299                 //  we tried to close stepslogger. Giving up now.
300             }
301             System.exit(statusCode);
302         }
303     }
304
305     private static int lastExitCode = 0;
306     private static ErrorManagementImplementation errorManagement = new ErrorManagementImplementati
    on();
307     protected static Logger LOG = LogManager.getLogger(BWAGobyArtifactScript.class);
308 }
309
```
